# TREM-1 deficiency attenuates RIPK3/MLKL-associated epithelial injury in *Klebsiella pneumoniae*-induced acute lung injury

**DOI:** 10.3389/fcell.2026.1974113

**Published:** 2026-10-13

**Authors:** Wei Zhang, Song Meng, Jiali Huang, Enfang Xiao, Yang Bai, Li Zhao, Kaifei Wang

**Affiliations:** 1 Department of Respiratory and Critical Care Medicine, First Medical Center, Chinese PLA General Hospital, Beijing, China; 2 Department of Respiratory and Critical Care Medicine, Mudanjiang Frist People’s Hospital, Mudanjiang, Heilongjiang, China; 3 The First Affiliated Hospital of Jinzhou Medical University, Jinzhou, Liaoning, China; 4 Department of Emergency, First Medical Center, Chinese PLA General Hospital, Beijing, China; 5 Department of Cardiology,First Medical Center, Chinese PLA General Hospital, Beijing, China; 6 Department of Respiratory and Critical Care Medicine, Peking University Shougang Hospital, Beijing, China

**Keywords:** acute lung injury, epithelial injury, *Klebsiella pneumoniae*, macrophage, mlkl, ripk3, TREM-1

## Abstract

**Background:**

*Klebsiella pneumoniae* (KP)-induced acute lung injury involves excessive myeloid inflammation, epithelial barrier disruption, and inflammatory cell death. Triggering receptor expressed on myeloid cells 1 (TREM-1) amplifies innate immune responses, whereas receptor-interacting protein kinase 3 (RIPK3)/mixed lineage kinase domain-like protein (MLKL) signaling contributes to membrane injury. Their relationship during bacterial lung infection remains incompletely understood.

**Methods:**

KP-induced acute lung injury was established in wild-type and *Trem1*-deficient mice, and GSK872 was administered prophylactically to inhibit RIPK3. Lung histopathology, inflammatory mediators, and RIPK3/MLKL activation were assessed. Two human lung single-cell RNA-sequencing datasets containing non-fibrotic and fibrotic tissues were analyzed as cross-disease, hypothesis-generating reference cohorts. *In vitro*, conditioned medium from KP-stimulated THP-1-derived macrophage-like cells was applied to BEAS-2B epithelial cells; macrophage TREM-1 was blocked with LP17, and epithelial RIPK3 was silenced using siRNA.

**Results:**

KP challenge increased pulmonary TREM-1 abundance and was accompanied by histopathological injury and altered inflammatory mediator concentrations. *Trem1* deficiency reduced these responses together with RIPK3 and MLKL phosphorylation. Prophylactic GSK872 administration produced changes in the same direction. In the human fibrosis reference cohorts, *TREM1* was concentrated in inflammatory myeloid populations, and ligand-receptor inference identified candidate macrophage-to-epithelial communication pathways. *In vitro*, KP increased TREM-1 and inflammatory mediator release from macrophage-like cells. Their conditioned medium induced epithelial membrane injury, RIPK3/MLKL phosphorylation, and loss of E-cadherin and ZO-1; these effects were attenuated by macrophage LP17 pretreatment or epithelial RIPK3 knockdown.

**Conclusion:**

*Trem1* deficiency attenuated KP-induced acute lung injury and was associated with reduced pulmonary inflammation and RIPK3/MLKL activation. The combined findings support a functional relationship between macrophage TREM-1-dependent inflammatory activation and epithelial RIPK3/MLKL-associated injury, rather than a fully established linear signaling axis. This intercellular injury network warrants further mechanistic and post-infection therapeutic evaluation.

## 1 Introduction

Acute lung injury (ALI) and its severe clinical manifestation, acute respiratory distress syndrome (ARDS), are characterized by diffuse pulmonary inflammation, disruption of the alveolar-capillary barrier, increased vascular permeability, and impaired gas exchange (Matth et al., 2024; Han and Mallampalli, 2015). Severe bacterial pneumonia and sepsis are major precipitating factors for ALI and ARDS (Han and Mallampalli, 2015; Nam et al., 2019). Despite advances in antimicrobial therapy, mechanical ventilation, and supportive care, effective molecularly targeted treatments remain limited. A better understanding of how pathogen-induced myeloid inflammation is coupled to structural epithelial injury may therefore reveal new therapeutic opportunities.

Dysregulated innate immune activation is a central feature of bacterial lung injury. Triggering receptor expressed on myeloid cells 1 (TREM-1) is an immunoglobulin superfamily receptor expressed predominantly on neutrophils and monocytes/macrophages (Bouchon et al., 2000; Bouchon et al., 2001). Through cooperation with pattern-recognition receptor signaling, TREM-1 amplifies the production of inflammatory cytokines and chemokines during microbial infection (Yuan et al., 2017; Zhong et al., 2023a). Experimental studies have shown that genetic deletion or pharmacological blockade of TREM-1 can alleviate excessive pulmonary inflammation and tissue injury (Bouchon et al., 2001; Yuan et al., 2017). However, because TREM-1 also participates in antimicrobial host responses, its contribution to lung injury must be evaluated in the context of specific pathogens and downstream tissue-damaging pathways.

Regulated inflammatory cell death is another important component of infection-induced lung injury. Necroptosis is mediated principally by receptor-interacting protein kinase 3 (RIPK3) and mixed lineage kinase domain-like protein (MLKL). Following phosphorylation by RIPK3, MLKL oligomerizes and translocates to cellular membranes, resulting in membrane permeabilization and the release of damage-associated molecular patterns (Morgan and Kim, 2022). Activation of RIPK3/MLKL signaling has been associated with epithelial and endothelial injury, inflammatory amplification, and adverse outcomes in experimental lung injury and human ARDS (Wang et al., 2016; Shashaty et al., 2019; Huang et al., 2021). Nevertheless, RIPK3 can also regulate inflammatory responses independently of MLKL-mediated membrane disruption. Consequently, increased RIPK3 and MLKL phosphorylation should be interpreted as evidence of necroptosis-associated signaling unless the execution of necroptotic cell death is demonstrated directly.

Although TREM-1-mediated inflammatory amplification and RIPK3/MLKL-associated injury have each been implicated in acute lung injury, the relationship between these processes remains unclear. Previous evidence indicates that TREM-1 can promote RIPK3/MLKL-dependent necroptosis in activated macrophages (Zhong et al., 2023b). It remains uncertain, however, whether TREM-1-dependent activation of myeloid cells promotes RIPK3/MLKL-associated injury in neighboring pulmonary epithelial cells. In particular, the cellular source of the relevant inflammatory mediators and the potential contribution of macrophage-epithelial communication have not been fully defined.

Single-cell RNA sequencing provides an opportunity to localize *TREM1* expression to specific pulmonary cell populations and to generate hypotheses regarding ligand-receptor communication. In this study, the four model systems served distinct purposes: the mouse KP model assessed *in vivo* associations during acute bacterial injury; prophylactic GSK872 tested the involvement of RIPK3-related signaling; the non-fibrotic and fibrotic human lung datasets provided cross-disease, hypothesis-generating cellular context; and the conditioned-medium system functionally examined whether soluble products from KP-stimulated macrophage-like cells could induce epithelial injury. The human fibrosis datasets were not treated as direct models of KP infection or ARDS, and computationally inferred communication was not interpreted as proof of causal signaling.

Accordingly, we investigated the effects of *Trem1* deficiency in a *Klebsiella pneumoniae*-induced mouse model of acute lung injury and evaluated whether these effects were accompanied by changes in pulmonary RIPK3/MLKL activation. Pharmacological RIPK3 inhibition was used to assess the functional relevance of this signaling pathway. We further examined *TREM1*-high macrophage states and predicted macrophage–epithelial interactions in human lung single-cell datasets. Finally, conditioned medium from KP-infected macrophage-like cells, TREM-1 blockade, and epithelial RIPK3 knockdown were used to determine whether macrophage TREM-1 activation was associated with RIPK3/MLKL signaling, membrane injury, and junctional protein loss in epithelial cells.

## 2 Materials and methods

### 2.1 Animals and experimental design

Eight-week-old male C57BL/6 J wild-type (WT) and *Trem1*-deficient (*Trem1*−/−) mice were obtained from Beijing Vital River Laboratory Animal Technology Co., Ltd. (Beijing, China). The *Trem1*-deficient mice were maintained on a C57BL/6 J background, and their genotypes were confirmed before experimentation. Animals were housed under specific pathogen-free conditions at 22 ± 2 °C and 50%–60% relative humidity under a 12-h light/dark cycle, with free access to food and water. Male mice were used to reduce sex-related biological variability; the restriction to one sex is acknowledged as a limitation.

Mice were randomly allocated to the WT + PBS, WT + KP, *Trem1* ^−/−^ + KP, and WT + KP + GSK872 groups, with five mice per group, using a computer-generated randomization sequence. Histological assessment and image quantification were performed by investigators blinded to group allocation.

For prophylactic inhibition of RIPK3, GSK872 (Selleck Chemicals, Houston, TX, USA; S8465) was administered intraperitoneally at 10 mg/kg 1 h before KP challenge. GSK872 was prepared according to the manufacturer’s instructions. Animals were euthanized 24 h after KP administration, and blood, bronchoalveolar lavage fluid (BALF), and lung tissues were collected for subsequent analyses.

All animal procedures were reviewed and approved by the Laboratory Animal Welfare and Ethics Committee of the First Medical Center, Chinese PLA General Hospital. No numerical approval code was assigned by the committee at the time of approval. The experiments were conducted in accordance with relevant institutional and national guidelines for the care and use of laboratory animals and are reported in accordance with the ARRIVE 2.0 guidelines.

### 2.2 Bacterial culture and preparation

*Klebsiella pneumoniae subsp. Pneumoniae* ATCC 43816 was purchased from the American Type Culture Collection (ATCC, Manassas, VA, USA). Bacteria were cultured in Luria–Bertani broth at 37 °C with shaking at 200 rpm until the logarithmic growth phase. Bacterial density was estimated by measuring optical density at 600 nm and was verified by serial dilution and colony counting on Luria–Bertani agar.

Bacteria were harvested by centrifugation at 4,000 × g for 10 min, washed twice with sterile PBS, and resuspended at the required concentration. For heat-killed KP experiments, bacterial suspensions were incubated at 65 °C for 30 min. Complete loss of bacterial viability was confirmed by plating the treated suspension on Luria–Bertani agar and verifying the absence of colony formation after overnight incubation.

### 2.3 Histological and immunohistochemical analyses

Lung tissues were fixed in 4% paraformaldehyde fixative solution (Beyotime Biotechnology, Shanghai, China; P0099), embedded in paraffin, and sectioned at a thickness of 5 μm. Sections were subjected to hematoxylin and eosin staining according to standard procedures. Representative images were acquired at ×100 and ×400 magnification.

Pulmonary injury was evaluated according to alveolar septal thickening, inflammatory cell infiltration, interstitial or alveolar edema, hemorrhage, and disruption of alveolar architecture. Five randomly selected, non-overlapping fields were evaluated for each animal by investigators blinded to group allocation.

For immunohistochemistry, sections were deparaffinized, rehydrated, subjected to heat-mediated antigen retrieval, and incubated overnight at 4 °C with antibodies against TREM-1 (Proteintech, Rosemont, IL, USA; 11791-1-AP), phosphorylated RIPK3 at Ser232 (Proteintech; 87148-1-RR), or phosphorylated MLKL at Ser345 (Cell Signaling Technology, Danvers, MA, USA; 37,333). After incubation with the appropriate horseradish peroxidase-conjugated secondary antibodies, immunoreactivity was visualized using a DAB chromogenic detection system and counterstained with hematoxylin.

### 2.4 BALF collection and inflammatory mediator measurement

After euthanasia, the trachea was exposed and cannulated. The lungs were lavaged three times with 0.8 mL of ice-cold sterile PBS. The recovered fluid was centrifuged at 500 *g* for 10 min at 4 °C, and the supernatant was collected and stored at −80 °C until analysis.

Blood samples were allowed to clot at room temperature and centrifuged at 1,500 × g for 15 min to obtain serum. Concentrations of soluble TREM-1, CRP, CXCL1, TNF-α, IL-1β, IL-6, IFN-γ, IL-2, IL-10, and IL-4 in serum and BALF were measured using commercial ELISA kits according to the manufacturers’ protocols. Absorbance was measured using a microplate reader, and analyte concentrations were calculated from the corresponding standard curves.

### 2.5 Cell culture, authentication, and *mycoplasma* testing

Human THP-1 monocytic leukemia cells were obtained from ATCC (TIB-202). Cells were maintained in RPMI-1640 medium (Gibco, Thermo Fisher Scientific, Waltham, MA, USA; 11875093) supplemented with 10% fetal bovine serum (Biological Industries, Beit HaEmek, Israel; 04–001-1A) and 1% penicillin–streptomycin (Gibco; 15140122).

Human BEAS-2B bronchial epithelial cells were obtained from ATCC and maintained in high-glucose DMEM (Gibco; 11,965,092) supplemented with 10% fetal bovine serum and 1% penicillin–streptomycin. Both cell lines were cultured at 37 °C in a humidified incubator containing 5% CO_2_.

### 2.6 THP-1 differentiation and KP stimulation

THP-1 cells were seeded at the required density and treated with 100 nM phorbol 12-myristate 13-acetate (PMA; Sigma-Aldrich, St. Louis, MO, USA; P8139) for 24 h. Cells were subsequently washed with PBS and rested in PMA-free complete medium for an additional 24 h to induce a macrophage-like phenotype.

Macrophage-like differentiation was evaluated by measuring CD68 and CD11b expression. Differentiated THP-1 cells were exposed to live KP at multiplicities of infection of 10 or 100 for the indicated periods. Mock-treated cells underwent the same medium-replacement procedure without bacterial exposure. Heat-killed KP was applied at an equivalent bacterial concentration.

### 2.7 Quantitative real-time PCR

Total RNA was extracted using TRIzol reagent (Invitrogen, Thermo Fisher Scientific; 15596026). RNA concentration and purity were assessed using a NanoDrop spectrophotometer. Equal amounts of RNA were reverse-transcribed into complementary DNA using the PrimeScript RT Reagent Kit with gDNA Eraser (Takara Bio, Shiga, Japan; RR047A).

Quantitative real-time PCR was performed using TB Green Premix Ex Taq II (Takara Bio; RR820A) on a QuantStudio real-time PCR system (Applied Biosystems, Thermo Fisher Scientific). Relative expression levels of *TREM1*, *CD68*, and *ITGAM*/*CD11B* were calculated using the 2^−^ΔΔCt method and normalized to *GAPDH*. Primer sequences are provided in Supplementary Table S1. Each reaction was performed in triplicate, and experiments were repeated in three independent biological replicates.

### 2.8 Flow-cytometric analysis of cell-surface TREM-1

After the indicated treatments, THP-1-derived macrophage-like cells were collected and washed with PBS containing 1% bovine serum albumin. Cells were incubated with PE-conjugated anti-human CD354/TREM-1 antibody (BioLegend, San Diego, CA, USA; 314,906) or the corresponding isotype control for 30 min at 4 °C in the dark.

Cells were washed twice and analyzed using a BD FACSCanto II flow cytometer (BD Biosciences, San Jose, CA, USA). Cellular debris and doublets were excluded using forward- and side-scatter parameters. Surface TREM-1 abundance was expressed as mean fluorescence intensity. Data were analyzed using FlowJo software.

### 2.9 Measurement of macrophage-derived inflammatory mediators

Culture supernatants from THP-1-derived macrophage-like cells were collected after the indicated treatments and centrifuged at 500 *g* for 10 min to remove cellular debris. Concentrations of soluble TREM-1, TNF-α, and IL-1β were measured using commercial human ELISA kits according to the manufacturers’ instructions. Cytokine concentrations were calculated from the corresponding standard curves. Experiments were performed in three independent biological replicates.

### 2.10 TREM-1 blockade and conditioned-medium preparation

For TREM-1 blockade, differentiated THP-1-derived macrophage-like cells were pretreated for 1 h with LP17, a synthetic TREM-1 inhibitory peptide with the sequence LQVTDSGLYRCVIYHPP, at 100 ng/mL before KP exposure.

Macrophage-like cells were infected with KP at a multiplicity of infection of 10 for 2 h, washed three times with sterile PBS, and cultured for an additional 18 h in fresh medium containing 100 μg/mL gentamicin (Gibco; 15750060). Uninfected macrophage-like cells underwent the same washing and medium-replacement procedures.

Conditioned medium was collected after 18 h, centrifuged at 1,000 × g for 10 min to remove cellular debris, and passed through a sterile 0.22-μm polyethersulfone syringe filter (Millex-GP, MilliporeSigma, Burlington, MA, USA; SLGP033RS). The filtered conditioned medium was immediately applied to BEAS-2B cells.

BEAS-2B cells were treated for 24 h with fresh control medium, conditioned medium from unstimulated macrophage-like cells (Mφ-CM), conditioned medium from KP-infected macrophage-like cells (Mφ-KP-CM), or conditioned medium from LP17-pretreated, KP-infected macrophage-like cells (LP17 + Mφ-KP-CM). Medium composition and gentamicin concentrations were maintained consistently across conditioned-medium groups.

### 2.11 Assessment of epithelial cell injury

Morphological changes in BEAS-2B cells were recorded using phase-contrast microscopy after 24 h of conditioned-medium exposure.

Cell death and loss of plasma membrane integrity were evaluated using the BD Pharmingen FITC Annexin V Apoptosis Detection Kit I (BD Biosciences; 556,547) according to the manufacturer’s protocol. Samples were analyzed using a BD FACSCanto II flow cytometer, and data were processed using FlowJo software. Annexin V^−^/PI^−^, Annexin V^+^/PI^−^, Annexin V^+^/PI^+^, and Annexin V^−^/PI^+^ populations were quantified separately. Because PI positivity indicates loss of plasma membrane integrity but is not specific to necroptosis, the results were interpreted as evidence of membrane injury.

Lactate dehydrogenase release into the culture supernatant was measured using the CytoTox 96 Non-Radioactive Cytotoxicity Assay (Promega, Madison, WI, USA; G1780). LDH release was calculated relative to spontaneous-release and maximum-release controls according to the manufacturer’s instructions. Experiments were performed in three independent biological replicates.

### 2.12 Western blot analysis

Total proteins were extracted from mouse lung tissues, THP-1-derived macrophage-like cells, and BEAS-2B cells using RIPA lysis and extraction buffer (Thermo Fisher Scientific; 89,900) supplemented with Halt Protease and Phosphatase Inhibitor Cocktail (Thermo Fisher Scientific; 78,440). Protein concentrations were measured using the Pierce BCA Protein Assay Kit (Thermo Fisher Scientific; 23,225).

Equal amounts of protein were separated by SDS-PAGE and transferred onto Immobilon-P PVDF membranes (MilliporeSigma; IPVH00010). Membranes were blocked with 5% bovine serum albumin for 1 h at room temperature.

For mouse lung samples, membranes were incubated overnight at 4 °C with antibodies against TREM-1 (Proteintech; 11791-1-AP; 1:1,000), phosphorylated RIPK3 at Ser232 (Proteintech; 87148-1-RR; 1:2,000), RIPK3 (Proteintech; 29080-1-AP; 1:1,000), phosphorylated MLKL at Ser345 (Cell Signaling Technology; 37,333; 1:1,000), MLKL (Proteintech; 66675-1-Ig; 1:2,000), or β-actin (Proteintech; 66009-1-Ig; 1:5,000).

For human cell samples, membranes were incubated with antibodies against TREM-1 (Proteintech; 30496-1-AP; 1:1,000), phosphorylated RIPK3 at Ser227 (Cell Signaling Technology; 93,654; 1:1,000), RIPK3 (Proteintech; 29080-1-AP; 1:1,000), phosphorylated MLKL at Ser358 (Proteintech; 82090-2-RR; 1:10,000), MLKL (Proteintech; 66675-1-Ig; 1:2,000), E-cadherin (Proteintech; 20874-1-AP; 1:5,000), ZO-1 (Proteintech; 21773-1-AP; 1:2,000), or β-actin (Proteintech; 66009-1-Ig; 1:5,000).

After washing, membranes were incubated with the appropriate horseradish peroxidase-conjugated secondary antibodies for 1 h at room temperature. Signals were developed using SuperSignal West Pico PLUS Chemiluminescent Substrate (Thermo Fisher Scientific; 34,580) and quantified using ImageJ. Phosphorylated RIPK3 and MLKL were normalized to their corresponding total protein levels, whereas other proteins were normalized to β-actin. Western blot analyses were performed using three independent biological replicates unless otherwise specified.

### 2.13 siRNA-mediated *RIPK3* knockdown

*RIPK3*-targeting siRNAs and the corresponding negative control siRNA were synthesized by GenePharma Co., Ltd. (Shanghai, China). BEAS-2B cells were transfected with 50 nM siRNA using Lipofectamine 3000 Transfection Reagent (Thermo Fisher Scientific; L3000015) according to the manufacturer’s protocol.

The *RIPK3*-targeting sequences were si-RIPK3-1, 5′-TTG​GAC​CCA​GAG​CTG​TTA​TTT-3′, and si-RIPK3-2, 5′-GTC​TCC​GAG​GTA​AAG​CAT​TAT-3′. *RIPK3* knockdown efficiency was evaluated by Western blotting 48 h after transfection. The siRNA exhibiting the greatest knockdown efficiency was selected for subsequent experiments. Transfected cells were exposed to the indicated conditioned media for 24 h before LDH, Western blotting, and immunofluorescence analyses.

### 2.14 Immunofluorescence staining

BEAS-2B cells grown on glass coverslips were fixed with 4% paraformaldehyde fixative solution (Beyotime Biotechnology; P0099) for 15 min, permeabilized with 0.1% Triton X-100 for 10 min, and blocked with 5% goat serum for 1 h at room temperature.

Cells were incubated overnight at 4 °C with an anti-E-cadherin antibody (Proteintech; 20874-1-AP; 1:200), followed by an Alexa Fluor 488-conjugated secondary antibody for 1 h at room temperature. Nuclei were counterstained with DAPI. Images were acquired using a Zeiss LSM 880 confocal laser-scanning microscope (Carl Zeiss, Oberkochen, Germany) under identical acquisition settings across groups. Fluorescence intensity and distribution were evaluated using ImageJ.

### 2.15 Human lung single-cell RNA-sequencing datasets

Publicly available human lung single-cell RNA-sequencing datasets GSE122960 and GSE135893 were obtained from the Gene Expression Omnibus database. Both datasets contain lung tissues from non-fibrotic donors and patients with pulmonary fibrosis. GSE122960 included eight non-fibrotic and eight fibrotic lung samples, whereas GSE135893 included 10 non-fibrotic and 20 fibrotic lung samples. The combined source cohort therefore comprised 18 non-fibrotic and 28 fibrotic samples. These datasets were analyzed as human fibrotic lung-disease reference cohorts and were not considered direct models of acute bacterial lung injury.

All single-cell analyses were performed using R version 4.4.3 and Seurat version 5.1.0. The two source datasets contained 190,466 cells before application of the study-specific quality-control criteria. The number of detected genes, total transcript counts, and mitochondrial transcript proportion were evaluated for each cell. Cells with more than 200 and fewer than 2,500 detected genes and a mitochondrial transcript proportion below 5% were retained. After quality control, 151,296 cells remained for integration and downstream analysis.

Gene-expression data were normalized using the NormalizeData function with the standard log-normalization procedure. The top 2,000 highly variable genes were identified using the FindVariableFeatures function with the variance-stabilizing transformation (vst) method. The expression matrix was subsequently centered and scaled using the ScaleData function, with mitochondrial transcript proportion included as a regressed technical covariate.

### 2.16 Single-cell data preprocessing, integration, and cell annotation

Principal component analysis was performed using the scaled expression matrix of the highly variable genes. To reduce potential batch effects across datasets and samples, Harmony was applied for sample-level integration. The first 30 Harmony-corrected principal components were used for downstream dimensionality reduction and clustering.

A shared nearest-neighbor graph was constructed from the first 30 Harmony-corrected principal components using the FindNeighbors function. Cells were then clustered using the Louvain algorithm implemented in the FindClusters function at a resolution of 1.0. Uniform Manifold Approximation and Projection was used for two-dimensional visualization of the integrated dataset.

Major cellular populations were annotated according to the coordinated expression of established positive lineage markers reported in the literature. Seven major cellular compartments were identified: epithelial cells, myeloid cells, T/NK cells, B/plasma cells, endothelial cells, stromal cells, and mast cells. The distribution and expression of *TREM1* across the major cell populations and between non-fibrotic and fibrotic lung samples were visualized using the FeaturePlot and VlnPlot functions.

### 2.17 Myeloid subclustering and differential-expression analysis

Myeloid cells were extracted from the integrated object and independently reprocessed and reclustered using Harmony-corrected dimensions. Clusters showing clear non-myeloid-cell contamination based on lineage-marker expression were excluded before final annotation.

Six monocyte/macrophage states were annotated according to the coordinated expression of multiple positive marker genes: *TREM1*-high inflammatory macrophages expressing *TREM1*, *IL1B*, *TNF*, *CXCL8*, and *CCL3*; interferon-responsive macrophages expressing *CXCL10*, *ISG15*, *GBP1*, *IFIT1*, and *IFIT3*; transitional monocyte-derived macrophages expressing *VCAN*, *FCN1*, S100A8, S100A9, and *CD14*; homeostatic macrophages expressing *HSPA1A*, *HSP90AA1*, *BAG3*, *HSPA1B*, and *DNAJB1*; interstitial macrophages expressing *F13A1*, *LYVE1*, *SELENOP*, *FOLR2*, and *MRC1*; and alveolar macrophages expressing *NR1H3*, *FABP4*, *PPARG*, *MARCO*, and *INHBA*. Representative positive marker genes supporting the annotation of the six monocyte/macrophage states are summarized in Supplementary Table S2.

Cluster-enriched genes were identified using FindAllMarkers with the Wilcoxon rank-sum test. Genes with an adjusted *P* value < 0.05, an average log_2_ fold change >0.25, and expression in more than 25% of cells in the corresponding population were considered differentially expressed. Differences in macrophage-state proportions were evaluated using individual samples as biological replicates.

### 2.18 Pseudotime trajectory analysis

Pseudotime analysis was performed using Monocle 2. To reduce cell-number imbalance, macrophage populations were downsampled to a maximum of 1,000 cells per state. A CellDataSet object was constructed from raw count data, and highly variable genes were used as ordering genes. Dimensionality reduction was performed using DDRTree.

The trajectory root was assigned to the state enriched in transitional monocyte-derived macrophages because its monocyte-associated marker profile was considered biologically compatible with an earlier transcriptional state. This choice provided an interpretive orientation rather than independent proof of lineage direction. Changes in *TREM1* and other trajectory-associated genes were visualized along pseudotime, and the trajectory was interpreted as pseudotemporal ordering and state continuity rather than confirmed differentiation.

### 2.19 Cell-cell communication analysis

Cell-cell communication was inferred using CellChat version 1.6.0 and the official human CellChatDB ligand–receptor database. Non-fibrotic and fibrotic samples were analyzed separately before comparison. Cell populations were downsampled to a maximum of 1,000 cells per population where necessary to reduce computational imbalance.

Overexpressed ligands, receptors, and ligand–receptor interactions were identified using the standard CellChat workflow. Communication probabilities were calculated using the recommended permutation procedure. Global interaction numbers and strengths, as well as macrophage-to-epithelial signaling, were compared between non-fibrotic and fibrotic lungs. *TNF*, IL-1, *FAS*, *SPP1*, and galectin-associated interactions were subsequently examined as pathways of interest.

CellChat results were interpreted as predicted communication potential rather than direct evidence of ligand–receptor binding or causal signaling. *TNF*-TNFRSF1A and *IL1B*-*IL1R1* interactions were therefore considered hypothesis-generating findings that informed the subsequent conditioned-medium experiments.

### 2.20 Statistical analysis

Animal data are presented as mean ± standard deviation, with five animals per group. *In vitro* data are presented as mean ± standard deviation from three independent biological experiments. Technical replicates were averaged before analysis and were not treated as independent biological replicates.

Comparisons between two independent groups were performed using an unpaired, two-tailed Student’s t-test when the data satisfied the assumptions of normality and homogeneity of variance. Comparisons among three or more groups were performed using one-way analysis of variance followed by Tukey’s multiple-comparison test. Normality was assessed using the Shapiro–Wilk test, and homogeneity of variance was evaluated using the Brown-Forsythe test. When the assumptions for parametric testing were not satisfied, the Mann-Whitney U test was used for comparisons between two groups, whereas the Kruskal–Wallis test followed by Dunn’s multiple-comparison test was used for comparisons among three or more groups.

For single-cell composition analyses, individual human samples rather than individual cells were treated as biological replicates. Multiple-testing correction was performed using the Benjamini–Hochberg method where applicable. A two-sided *P* value < 0.05 was considered statistically significant. Statistical analyses were performed using GraphPad Prism nine and R version 4.4.3.

## 3 Results

### 3.1 TREM-1 expression is increased in *Klebsiella pneumoniae*-induced acute lung injury

To determine whether TREM-1 is involved in bacterial infection-induced lung injury, we established a *K. pneumoniae* (KP)-induced acute lung injury model in mice. Histological examination showed that the WT + PBS group maintained relatively normal alveolar architecture, with thin alveolar septa and minimal inflammatory cell infiltration. In contrast, WT mice challenged with KP exhibited histopathological alterations, including disruption of alveolar architecture, irregular alveolar spaces, interstitial edema, alveolar septal thickening, and inflammatory cell infiltration (Figure 1A). These findings confirmed the induction of acute inflammatory lung injury following intratracheal KP administration.

**FIGURE 1:**
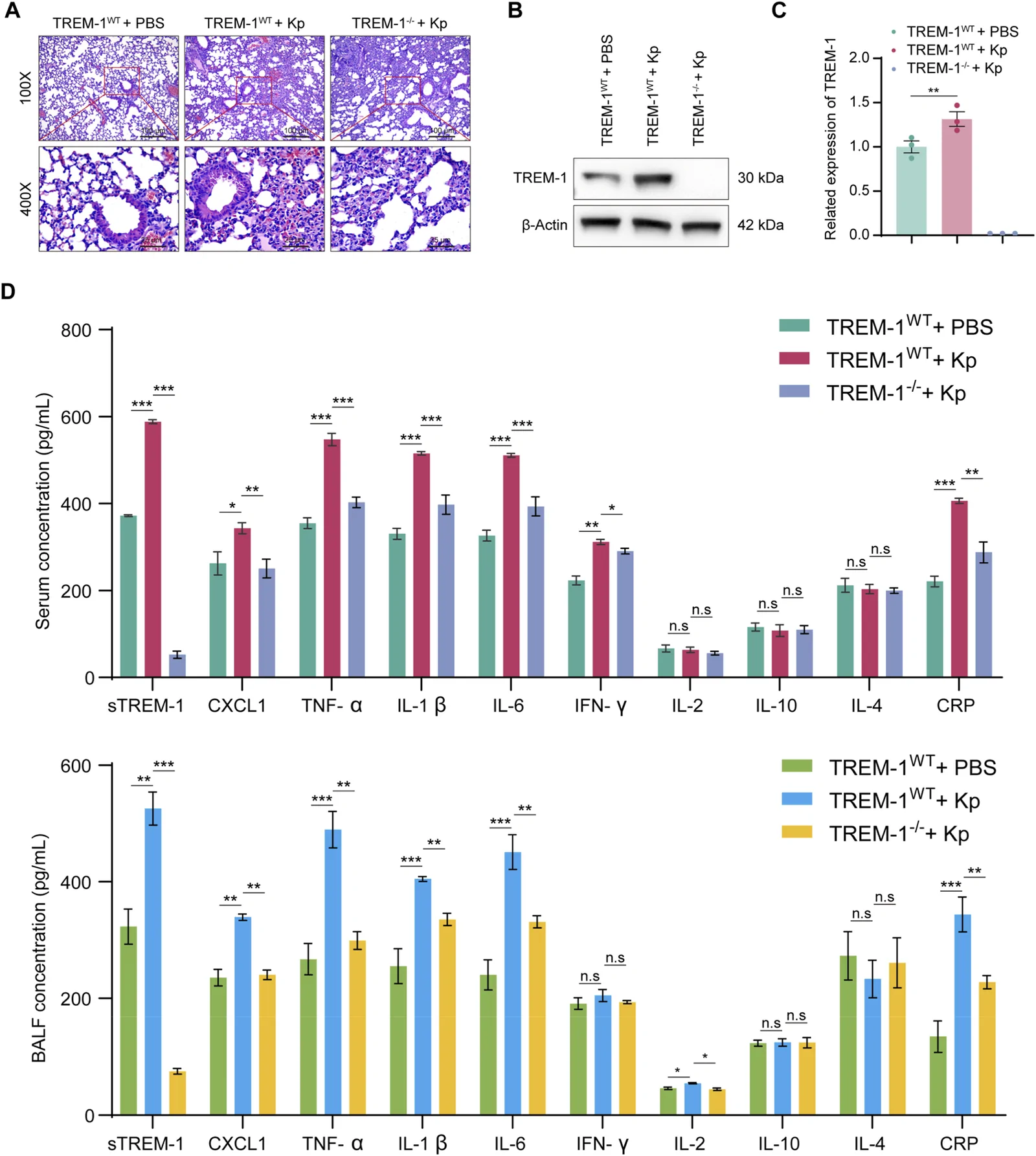
*Trem1* deficiency attenuates *Klebsiella pneumoniae*-induced acute lung injury and inflammatory responses. **(A)** Representative hematoxylin and eosin-stained lung sections from WT + PBS, WT + KP, and *Trem1* ^−/−^ + KP mice, showing alterations in alveolar architecture, septal thickness, interstitial edema, and inflammatory cell infiltration. Images were acquired at ×100 and ×400 magnification. Scale bars are shown in the images. **(B)** Representative Western blot images of TREM-1 and β-actin in lung tissues. **(C)** Densitometric quantification of TREM-1 protein abundance normalized to β-actin. **(D)** Concentrations of soluble TREM-1, CRP, CXCL1, TNF-α, IL-1β, IL-6, IFN-γ, IL-2, IL-10, and IL-4 in serum and bronchoalveolar lavage fluid, as measured by ELISA. Data are presented as mean ± SD; n = 5 mice per group. *P < 0.05, **P < 0.01, ***P < 0.001; n. s., not significant.

We next assessed pulmonary TREM-1 expression. Western blotting showed that TREM-1 protein abundance was increased in the lungs of WT + KP mice compared with WT + PBS controls. As expected, TREM-1 protein was undetectable in lung tissues from *Trem1* ^−/−^ + KP mice, consistent with the knockout genotype (Figures 1B,C). Soluble TREM-1 concentrations were also increased after KP challenge but remained low in *Trem1*-deficient mice (Figure 1D). Collectively, these findings indicate that KP-induced acute lung injury is accompanied by activation of the pulmonary TREM-1 response.

### 3.2 *Trem* ^*1*^ deficiency attenuates KP-induced lung injury and inflammatory responses

To evaluate the contribution of TREM-1 to KP-induced lung injury, histopathological and inflammatory changes were compared between WT and *Trem1*-deficient mice. Compared with the WT + KP group, *Trem1* ^−/−^ + KP mice exhibited relatively preserved alveolar architecture, reduced interstitial edema, less pronounced alveolar septal thickening, and decreased inflammatory cell infiltration (Figure 1A). These observations indicate that *Trem1* deficiency attenuates the structural lung injury induced by KP challenge.

KP administration increased CRP, CXCL1, TNF-alpha, IL-1beta, IL-6, and IFN-gamma concentrations in serum and BALF, consistent with systemic and pulmonary inflammatory activation. IL-2, IL-10, and IL-4 were analyzed separately as immune-regulatory mediators because their functions are context dependent and should not be interpreted uniformly as pro-inflammatory. *Trem1*−/− + KP mice showed lower concentrations of several inflammatory mediators than WT + KP mice, whereas changes in IL-2, IL-10, and IL-4 were smaller or not significant in several comparisons (Figure 1D). Thus, *Trem1* deficiency altered the mediator profile accompanying KP-induced injury rather than uniformly suppressing every measured cytokine.

### 3.3 *Trem* ^*1*^ deficiency is associated with reduced RIPK3/MLKL pathway activation

We next investigated whether the protective phenotype observed in *Trem1*-deficient mice was accompanied by altered RIPK3/MLKL signaling. KP challenge increased the phosphorylation of RIPK3 and MLKL in lung tissues, whereas the corresponding total protein levels showed no comparable increase (Figures 2A,B). This pattern was consistent with activation of the RIPK3/MLKL signaling pathway during KP-induced lung injury.

**FIGURE 2:**
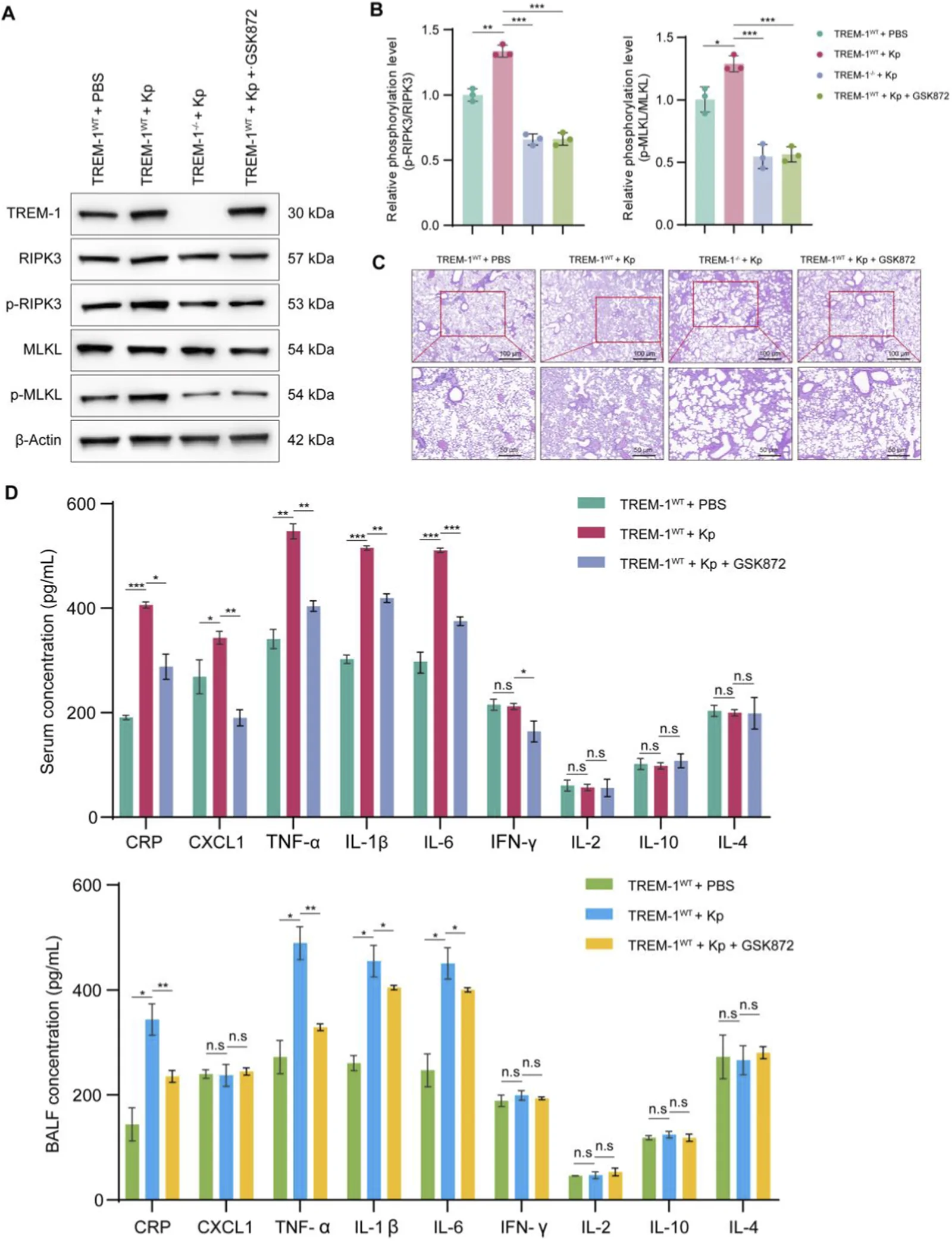
Prophylactic RIPK3 inhibition attenuates *Klebsiella pneumoniae*-induced RIPK3/MLKL pathway activation, lung injury, and inflammatory responses. **(A)** Representative Western blot images of TREM-1, RIPK3, p-RIPK3, MLKL, p-MLKL, and β-actin in lung tissues from the indicated groups. **(B)** Densitometric quantification of RIPK3 and MLKL phosphorylation, expressed as the p-RIPK3/RIPK3 and p-MLKL/MLKL ratios. **(C)** Representative hematoxylin and eosin-stained lung sections showing alterations in alveolar architecture, septal thickness, interstitial edema, and inflammatory cell infiltration following KP challenge and GSK872 administration. Images were acquired at ×100 and ×400 magnification. Scale bars are shown in the images. **(D)** Concentrations of CRP, CXCL1, TNF-α, IL-1β, IL-6, IFN-γ, IL-2, IL-10, and IL-4 in serum and bronchoalveolar lavage fluid, as measured by ELISA. Data are presented as mean ± SD; n = 5 mice per group. *P < 0.05, **P < 0.01, ***P < 0.001; n. s., not significant.

Compared with WT + KP mice, *Trem1* ^−/−^ + KP mice exhibited lower p-RIPK3/RIPK3 and p-MLKL/MLKL ratios (Figures 2A,B). These results indicate that *Trem1* deficiency is associated with reduced pulmonary RIPK3/MLKL pathway activation during bacterial lung injury. Because genetic or pharmacological inhibition of MLKL was not performed *in vivo*, these findings were interpreted as evidence of attenuated necroptosis-associated signaling rather than definitive quantification of necroptotic cell death.

### 3.4 Prophylactic RIPK3 inhibition attenuates KP-induced lung injury

To examine the functional contribution of RIPK3-related signaling, WT mice were treated with GSK872 1 h before KP challenge. GSK872 administration reduced the phosphorylation of RIPK3 and MLKL in lung tissues compared with the WT + KP group (Figures 2A,B), indicating effective suppression of the RIPK3/MLKL pathway under the experimental conditions.

Histological examination showed that GSK872-treated mice exhibited less pronounced alveolar disruption, interstitial edema, septal thickening, and inflammatory cell infiltration than untreated WT + KP mice (Figure 2C). In parallel, inflammatory mediator concentrations in serum and BALF were reduced following GSK872 administration (Figure 2D). The direction of these changes was similar to that observed in *Trem1*-deficient mice. These findings support the involvement of RIPK3-associated signaling in KP-induced inflammatory lung injury. Because GSK872 was administered before bacterial challenge, these data demonstrate a prophylactic protective effect rather than a therapeutic effect after disease establishment.

### 3.5 Human lung single-cell analysis identifies a *TREM1*-high inflammatory macrophage state in fibrosis-associated pulmonary remodeling

To characterize the cellular distribution of *TREM1* in diseased human lungs, we analyzed the GSE122960 and GSE135893 single-cell RNA-sequencing datasets, which contain lung tissues from non-fibrotic donors and patients with pulmonary fibrosis. These datasets were used as human lung-disease reference cohorts and were not considered direct models of acute bacterial lung injury.

Following quality control, integration, and unsupervised clustering, seven major cellular compartments were identified: epithelial cells, myeloid cells, T/NK cells, B/plasma cells, endothelial cells, stromal cells, and mast cells (Figures 3A,B). Expression of established lineage markers supported the assigned cellular annotations (Figure 3C).

**FIGURE 3:**
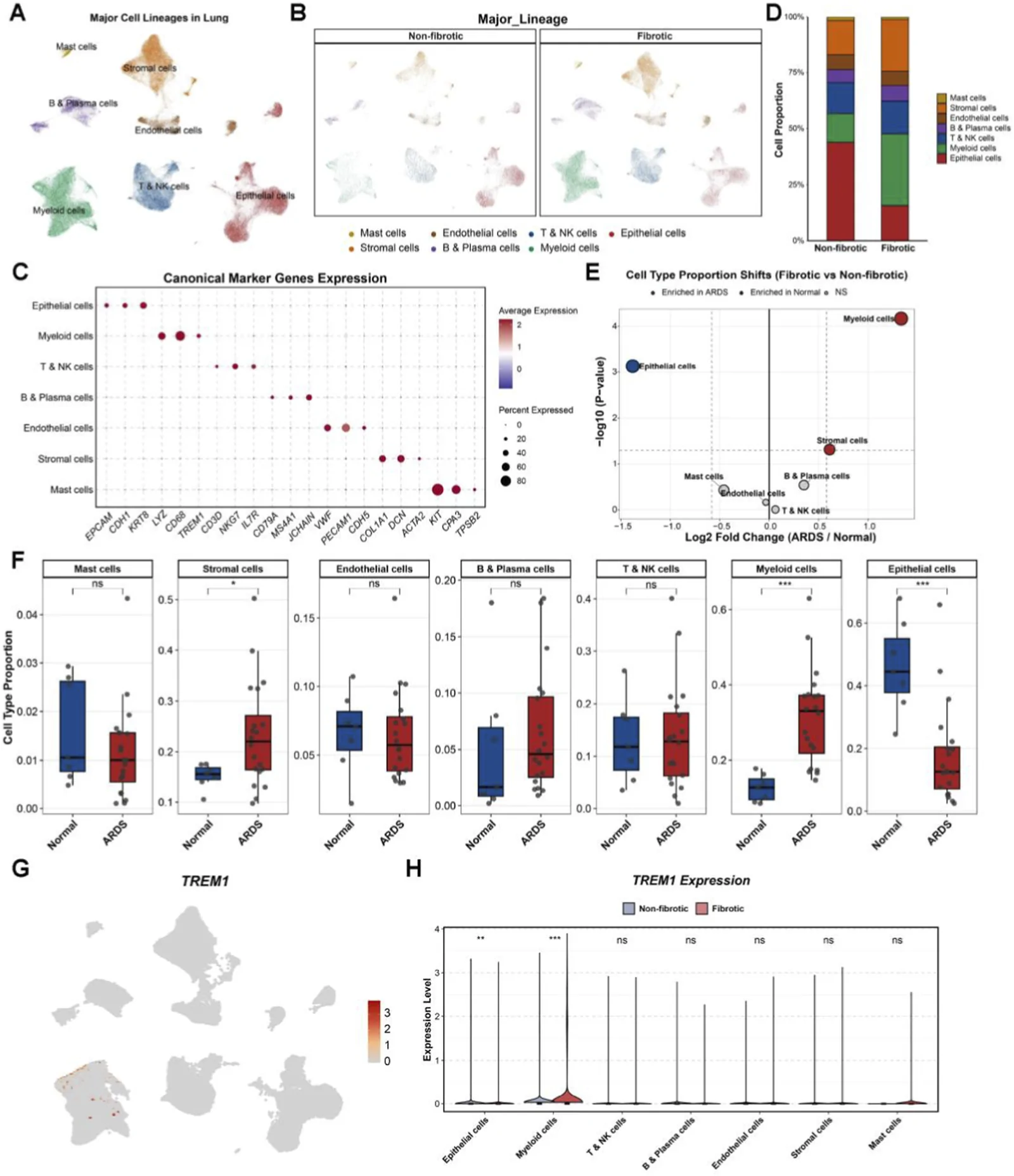
Single-cell transcriptomic landscape of non-fibrotic and fibrotic human lungs. **(A)** UMAP representation of integrated single-cell RNA-sequencing data from the GSE122960 and GSE135893 datasets, showing seven major lung cell compartments. **(B)** UMAP plots stratified by non-fibrotic and fibrotic lung samples. **(C)** Dot plot showing the expression of representative lineage markers and *TREM1* across the major cell compartments. Dot size represents the percentage of cells expressing each gene, and color intensity represents the average scaled expression level. **(D)** Stacked bar plots showing the relative proportions of the major cell compartments in non-fibrotic and fibrotic lungs. **(E)** Proportional-shift plot showing changes in cell-type abundance between fibrotic and non-fibrotic lungs. Dashed lines indicate the predefined thresholds of *P* < 0.05 and |log_2_ fold change| > 0.58. **(F)** Sample-level comparisons of the proportions of each major cell compartment between non-fibrotic and fibrotic lungs. Each point represents an individual lung sample. **(G)** UMAP feature plot showing the distribution of *TREM1* expression across the integrated lung single-cell dataset. **(H)** Violin plots showing *TREM1* expression across the major cell compartments, stratified by disease group. These pulmonary fibrosis datasets were analyzed as human lung-disease reference cohorts and do not represent acute bacterial lung injury or ARDS. Group comparisons were performed as described in Section 2.20. UMAP, uniform manifold approximation and projection. *P < 0.05, **P < 0.01, ***P < 0.001; n. s., not significant.

Analysis of cellular composition revealed extensive remodeling of fibrotic lungs relative to non-fibrotic controls (Figures 3D–F). Sample-level comparisons indicated an increased representation of myeloid and stromal populations and a reduced representation of epithelial cells in fibrotic lungs. These changes were consistent with the inflammatory infiltration, epithelial remodeling, and stromal expansion characteristic of pulmonary fibrosis. Because the analyzed samples were obtained from chronic fibrotic lung disease, these compositional differences should not be interpreted as infection- or ARDS-specific changes.

*TREM1* expression was predominantly localized to the myeloid compartment, with substantially lower expression in most lymphoid, stromal, endothelial, and epithelial populations (Figure 3G). Within the myeloid compartment, *TREM1* expression was higher in fibrotic lungs than in non-fibrotic controls (Figure 3H). Low-level *TREM1* transcripts were also detected in a subset of epithelial cells; however, the dominant cellular source remained the myeloid population. These findings identify inflammatory myeloid cells as the principal *TREM1*-expressing compartment in diseased human lungs.

We next re-extracted and reclustered the myeloid cells, identifying six monocyte/macrophage states: *TREM1*-high inflammatory macrophages, interferon-responsive macrophages, transitional monocyte-derived macrophages, homeostatic macrophages, interstitial macrophages, and alveolar macrophages (Figures 4A,B). Their identities were supported by the expression of established state-associated markers (Figure 4C). The *TREM1*-high inflammatory macrophage population co-expressed *TREM1*, *IL1B*, and *TNF*, indicating a transcriptional program associated with inflammatory mediator production.

**FIGURE 4:**
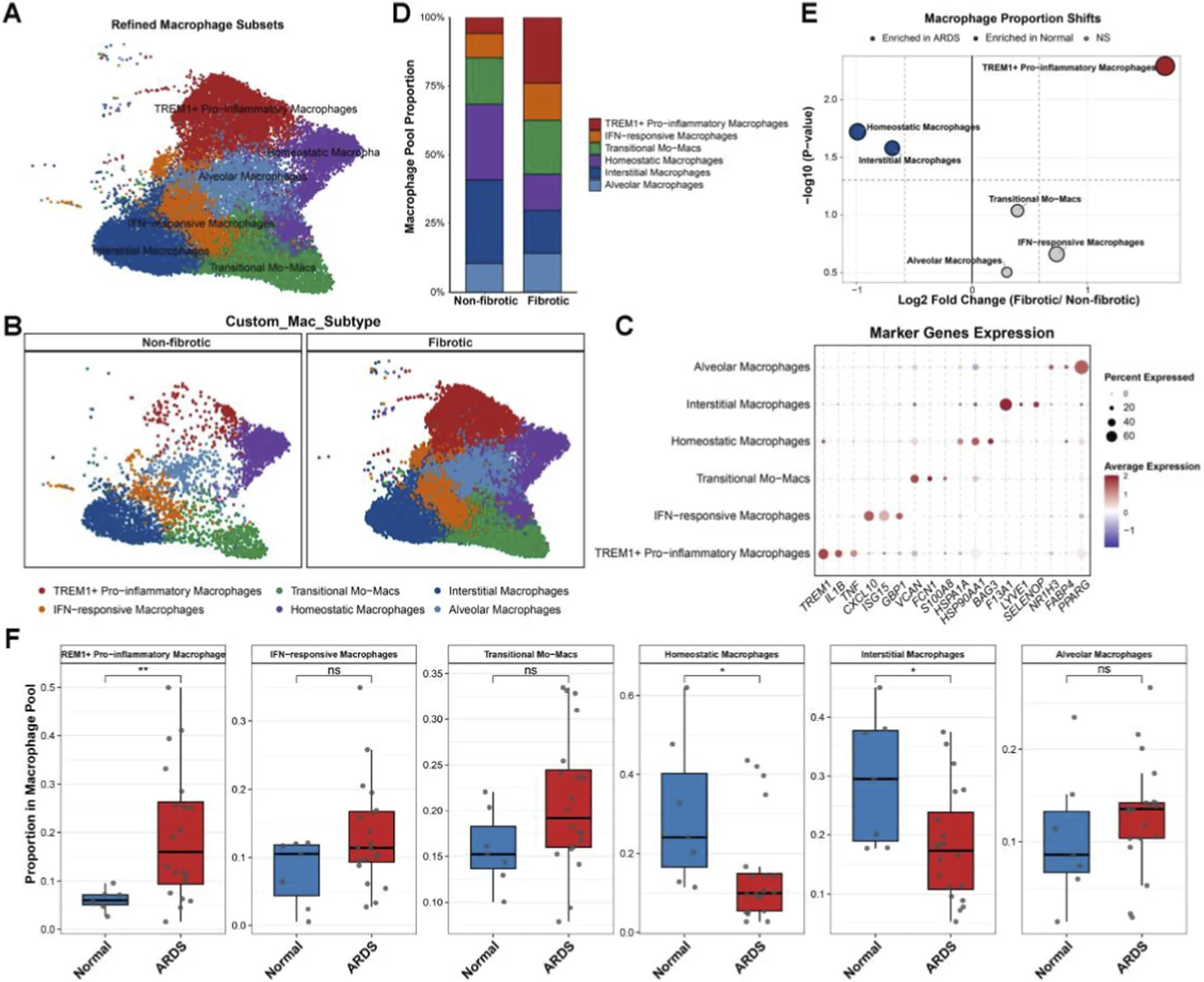
Macrophage heterogeneity in non-fibrotic and fibrotic human lungs. **(A)** UMAP representation of six monocyte/macrophage states identified after re-extraction and reclustering of the myeloid compartment: *TREM1*-high inflammatory macrophages, interferon-responsive macrophages, transitional monocyte-derived macrophages, homeostatic macrophages, interstitial macrophages, and alveolar macrophages. **(B)** UMAP plots stratified by non-fibrotic and fibrotic lung samples. **(C)** Dot plot showing the expression of representative marker genes across the six monocyte/macrophage states. Dot size represents the percentage of cells expressing each gene, and color intensity represents the average scaled expression level. **(D)** Stacked bar plots showing the relative proportions of the six states within the monocyte/macrophage compartment in non-fibrotic and fibrotic lungs. **(E)** Proportional-shift plot showing changes in the abundance of each state between fibrotic and non-fibrotic lungs. Dashed lines indicate the predefined thresholds of *P* < 0.05 and |log_2_ fold change| > 0.58. **(F)** Sample-level comparisons of the relative proportions of each monocyte/macrophage state between non-fibrotic and fibrotic lungs. Each point represents an individual lung sample. *P < 0.05; n. s., not significant.

Cell-composition analyses showed that *TREM1*-high inflammatory macrophages were enriched in fibrotic lungs, whereas homeostatic and interstitial macrophage populations were relatively reduced (Figures 4D–F). The proportions of transitional monocyte-derived, interferon-responsive, and alveolar macrophage populations showed less consistent differences between groups. These findings suggest that pulmonary fibrosis is associated with a shift from homeostatic macrophage states toward a *TREM1*-high inflammatory state.

To explore transcriptional relationships among the macrophage populations, we performed pseudotime analysis using Monocle 2. The inferred ordering placed transitional monocyte-derived macrophages near the selected root and the *TREM1*-high inflammatory population toward a distal region (Figures 5A–D). *TREM1* expression increased along part of the pseudotemporal axis and was highest in the inflammatory state (Figure 5E). These results indicate transcriptional state continuity between monocyte-associated and *TREM1*-high inflammatory macrophage states. Because pseudotime does not directly establish lineage or temporal direction, the analysis was not interpreted as confirmed monocyte-to-macrophage differentiation.

**FIGURE 5:**
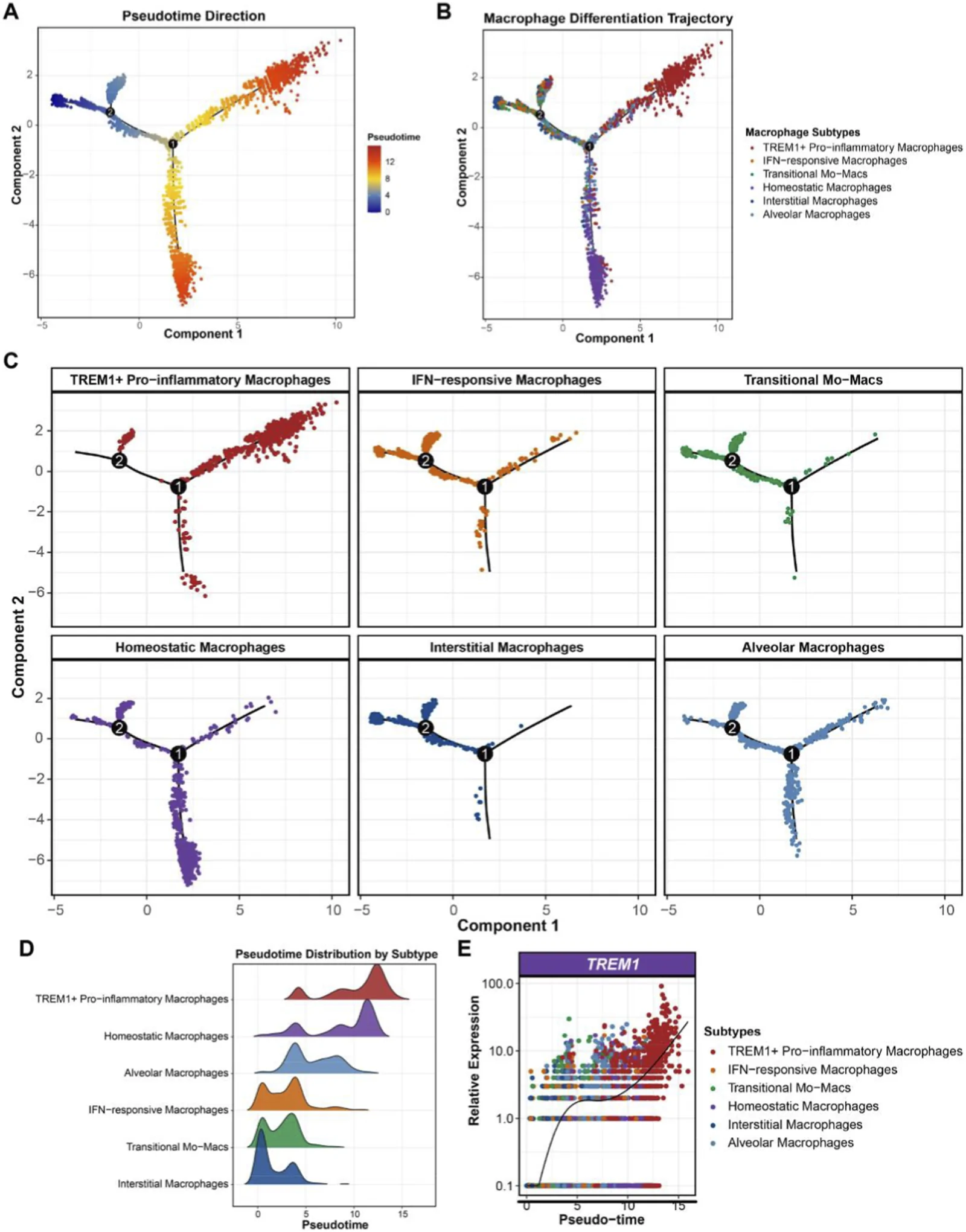
Pseudotime analysis suggests a potential transition toward a *TREM1*-high inflammatory macrophage state. **(A)** Monocle 2 trajectory of monocyte/macrophage cells, colored according to the six annotated cellular states. Each point represents an individual cell. **(B)** The inferred trajectory colored by pseudotime, with transitional monocyte-derived macrophages assigned as the root population. **(C)** Distribution of the six monocyte/macrophage states along the inferred pseudotemporal trajectory. **(D)** Density distributions showing the relative positioning of each cellular state along pseudotime. **(E)** Smoothed expression profile of *TREM1* along the pseudotemporal axis, showing increased expression toward the region enriched in *TREM1*-high inflammatory macrophages. The inferred trajectory represents transcriptional state continuity and does not establish cellular lineage, directionality, or confirmed differentiation.

### 3.6 KP induces TREM-1 expression and inflammatory activation in THP-1-derived macrophage-like cells

To determine whether KP directly promotes a TREM-1-associated inflammatory phenotype in human macrophage-like cells, THP-1 cells were differentiated using PMA. PMA stimulation increased the mRNA expression of the macrophage-associated markers *CD68* and *CD11B* (Figure 6A). Western blotting also showed increased *CD68* and CD11b protein abundance after PMA treatment (Figures 6B,C), supporting successful acquisition of a macrophage-like phenotype.

**FIGURE 6:**
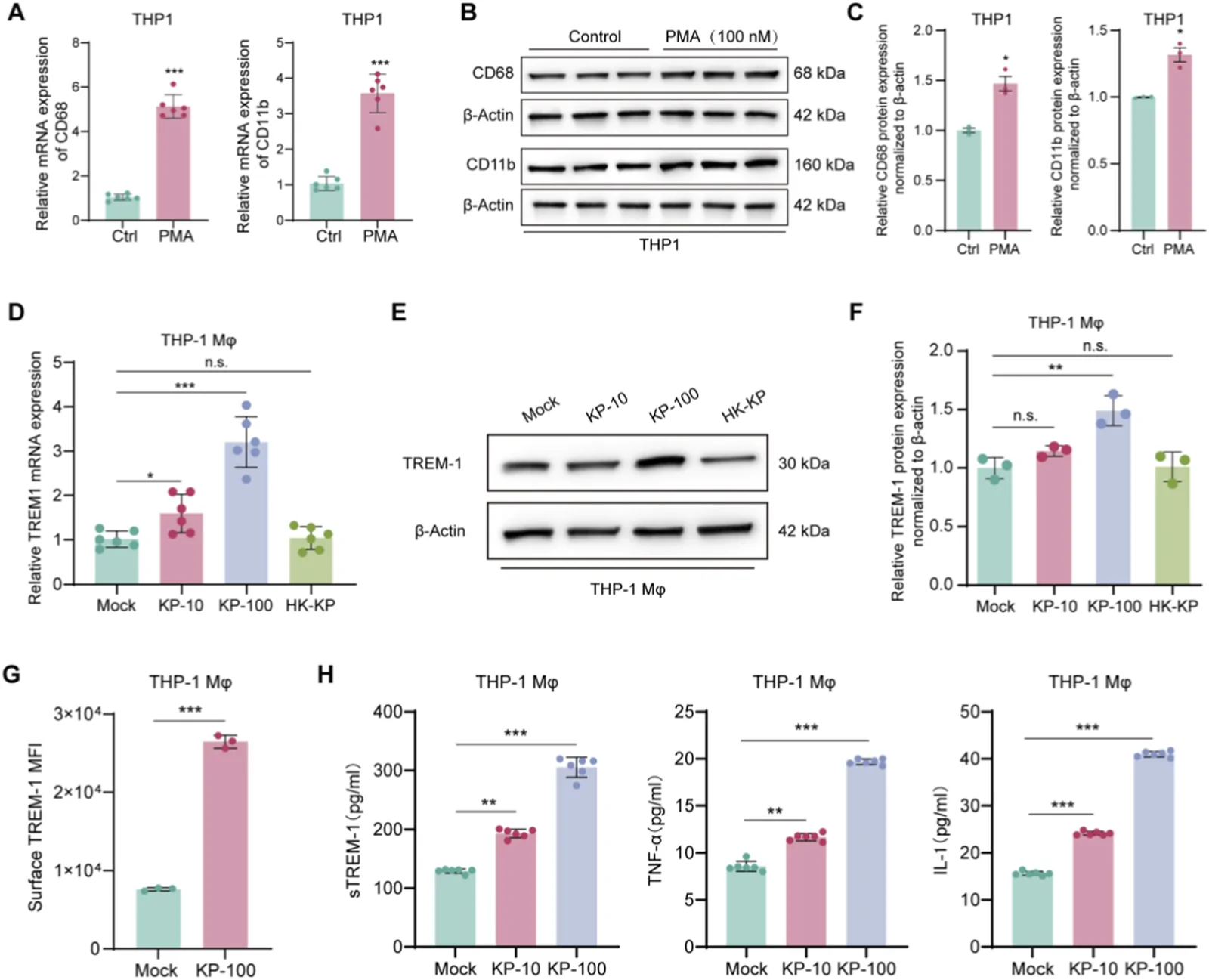
*Klebsiella pneumoniae* induces TREM-1 expression and inflammatory activation in THP-1-derived macrophage-like cells. **(A)** RT-qPCR analysis of the macrophage-associated markers *CD68* and *CD11B* in THP-1 cells before and after PMA-induced differentiation. **(B)** Representative Western blots showing *CD68* and CD11b protein expression. **(C)** Densitometric quantification of *CD68* and CD11b normalized to β-actin. **(D)** RT-qPCR analysis of *TREM1* expression in differentiated THP-1 cells treated with medium alone (Mock), live *K. pneumoniae* at a multiplicity of infection (MOI) of 10 or 100, or heat-killed *K. pneumoniae* (HK-KP). **(E)** Representative Western blots showing TREM-1 protein expression under the indicated conditions. **(F)** Densitometric quantification of TREM-1 normalized to β-actin. **(G)** Flow-cytometric analysis of cell-surface TREM-1 following infection with live *K. pneumoniae* at an MOI of 100. Surface expression is presented as mean fluorescence intensity (MFI). **(H)** Concentrations of soluble TREM-1 (sTREM-1), TNF-α, and IL-1β in culture supernatants from differentiated THP-1 cells exposed to medium alone or live *K. pneumoniae* at an MOI of 10 or 100, as determined by ELISA. Transcript levels were normalized to *GAPDH* and expressed relative to the corresponding control group. Data are presented as mean ± SD from three independent biological experiments. *P < 0.05, **P < 0.01, ***P < 0.001; n. s., not significant.

Live KP increased *TREM1* mRNA expression compared with the mock-treated group, with a more pronounced response observed at an MOI of 100. Heat-killed KP also increased *TREM1* expression, although the magnitude of the response differed from that induced by live bacteria (Figure 6D). Consistently, TREM-1 protein abundance was increased following KP exposure, particularly after stimulation with live bacteria (Figures 6E,F).

Flow-cytometric analysis further demonstrated an increase in cell-surface TREM-1 abundance after live KP exposure, as reflected by elevated mean fluorescence intensity (Figure 6G). In addition, KP increased the concentrations of soluble TREM-1, TNF-α, and IL-1β in culture supernatants (Figure 6H). These findings demonstrate that KP exposure induces TREM-1 expression and inflammatory mediator production in THP-1-derived macrophage-like cells.

### 3.7 Ligand-receptor analysis predicts communication between *TREM1*-high inflammatory macrophages and lung epithelial cells

To explore the potential communication between macrophage states and other cellular compartments in diseased human lungs, we performed CellChat analysis using the official human ligand-receptor database. The predicted global communication networks showed extensive interactions among macrophage, stromal, epithelial, and endothelial populations (Figures 7A,B). Analysis of incoming and outgoing interaction strengths indicated that macrophage and stromal populations represented prominent predicted signal senders, whereas epithelial, endothelial, and selected myeloid populations showed substantial predicted signal-receiving activity (Figure 7C).

**FIGURE 7:**
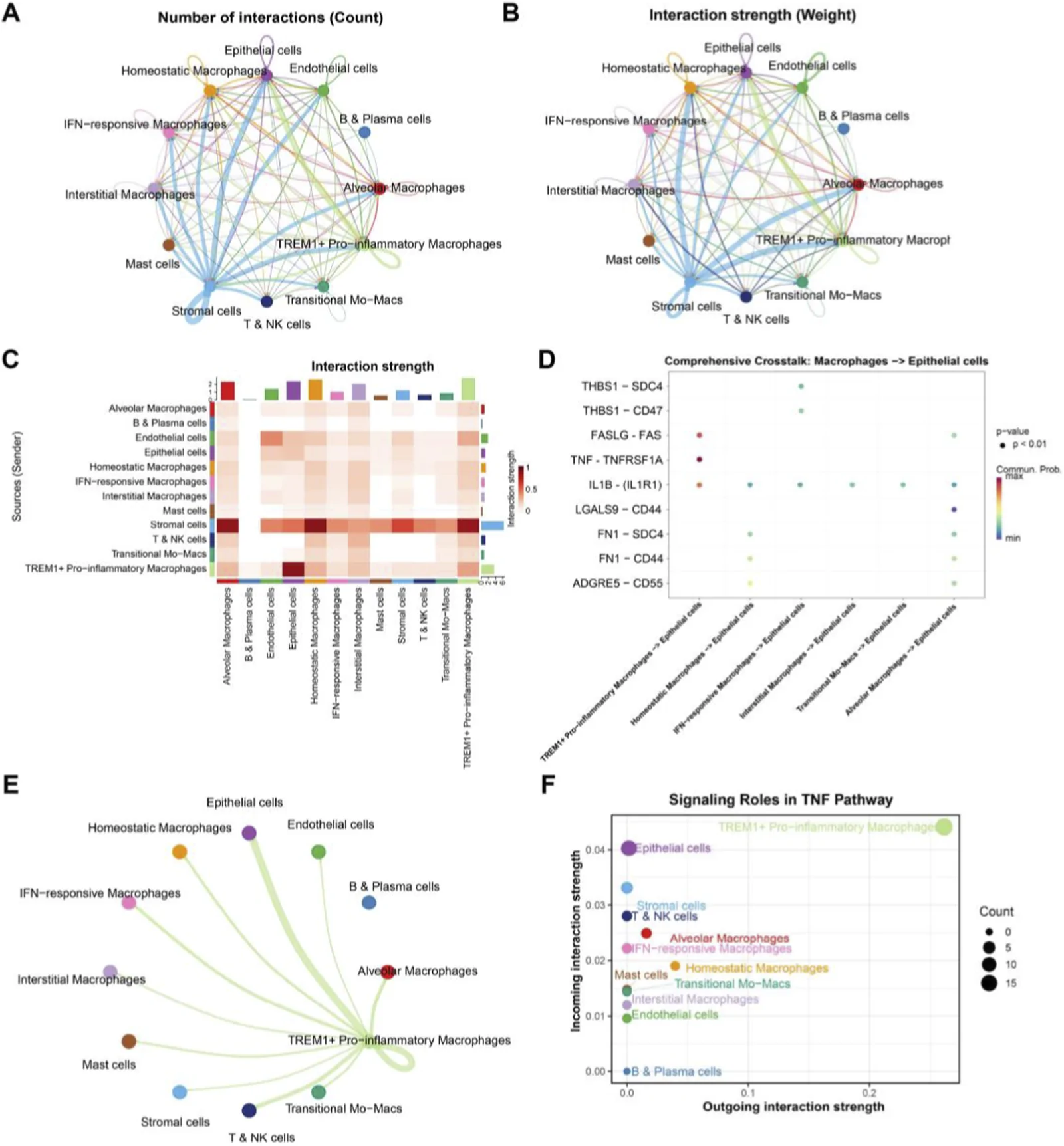
Predicted macrophage-epithelial ligand-receptor communication in fibrotic human lungs. **(A)** CellChat circle plot showing the inferred number of interactions among the major lung cell compartments and monocyte/macrophage states. **(B)** Circle plot showing the aggregate weights of the inferred ligand–receptor interactions. Edge width represents the predicted interaction strength. **(C)** Heatmap showing the predicted communication strength between sender and receiver populations. Rows represent signal senders, columns represent receivers, and the accompanying bar plots summarize total outgoing and incoming signaling strengths. **(D)** Dot plot showing selected ligand–receptor interactions predicted from the six monocyte/macrophage states toward epithelial cells, including *TNF*-TNFRSF1A, *FASLG*-FAS, *IL1B*-IL1R1, *SPP1*-CD44, and *LGALS9*-*CD44*. **(E)** Aggregated network of the predicted *TNF* signaling pathway, showing *TREM1*-high inflammatory macrophages as a prominent source of outgoing *TNF* signaling and epithelial and stromal populations as predicted recipients. **(F)** CellChat signaling-role plot for the *TNF* pathway. The horizontal and vertical axes represent predicted outgoing and incoming signaling strengths, respectively, and dot size represents the relative number of cells in each population. These analyses infer communication potential from coordinated ligand and receptor expression and do not demonstrate direct ligand secretion, receptor activation, or causal signaling. The pulmonary fibrosis datasets provide cross-disease supportive evidence and do not directly represent KP-induced acute lung injury.

We next focused on ligand–receptor interactions predicted from the six macrophage states toward epithelial cells. The *TREM1*-high inflammatory macrophage population showed relatively strong predicted interactions involving *TNF*-TNFRSF1A, *FASLG*-FAS, *IL1B*-IL1R1, *SPP1*-CD44, and *LGALS9*-CD44 (Figure 7D). *TNF*-TNFRSF1A and *FASLG*–*FAS* are associated with cell-death signaling, whereas *IL1B*-IL1R1, *SPP1*-CD44, and *LGALS9*-CD44 may contribute to inflammatory activation, epithelial remodeling, or altered cell adhesion. *IL1B*-IL1R1 signaling was also predicted from the interferon-responsive macrophage population.

Because ligand–receptor inference is based on coordinated expression rather than direct measurement of secreted proteins or receptor activation, these interactions were interpreted as predicted communication potential. In particular, the analysis did not establish that *TNF* or IL-1β directly activated epithelial RIPK3/MLKL signaling.

Focused analysis of the TNF pathway identified the *TREM1*-high inflammatory macrophage population as a prominent predicted source of outgoing TNF signaling, whereas epithelial and stromal populations showed substantial predicted incoming signaling activity (Figures 7E,F). These computational findings provided a rationale for testing whether soluble mediators released from KP-stimulated macrophage-like cells could induce injury-associated signaling in epithelial cells. However, because the single-cell datasets were derived from pulmonary fibrosis, the predicted communication network was considered cross-disease supportive evidence rather than a direct representation of KP-induced acute lung injury.

### 3.8 TREM-1-activated macrophage-like cells induce RIPK3/MLKL-associated epithelial injury and junctional disruption

To experimentally examine whether soluble mediators released from KP-stimulated macrophage-like cells could affect epithelial cells, conditioned medium from THP-1-derived macrophage-like cells was applied to BEAS-2B cells. BEAS-2B cells treated with fresh medium served as the normal control, whereas cells treated with conditioned medium from unstimulated or KP-infected macrophage-like cells were designated the Mφ-CM and Mφ-KP-CM groups, respectively.

BEAS-2B cells in the normal-control and Mφ-CM groups maintained relatively intact morphology. In contrast, Mφ-KP-CM exposure induced cell shrinkage, reduced adherence, and disruption of monolayer integrity (Figure 8A), indicating epithelial injury in response to factors present in the conditioned medium.

**FIGURE 8:**
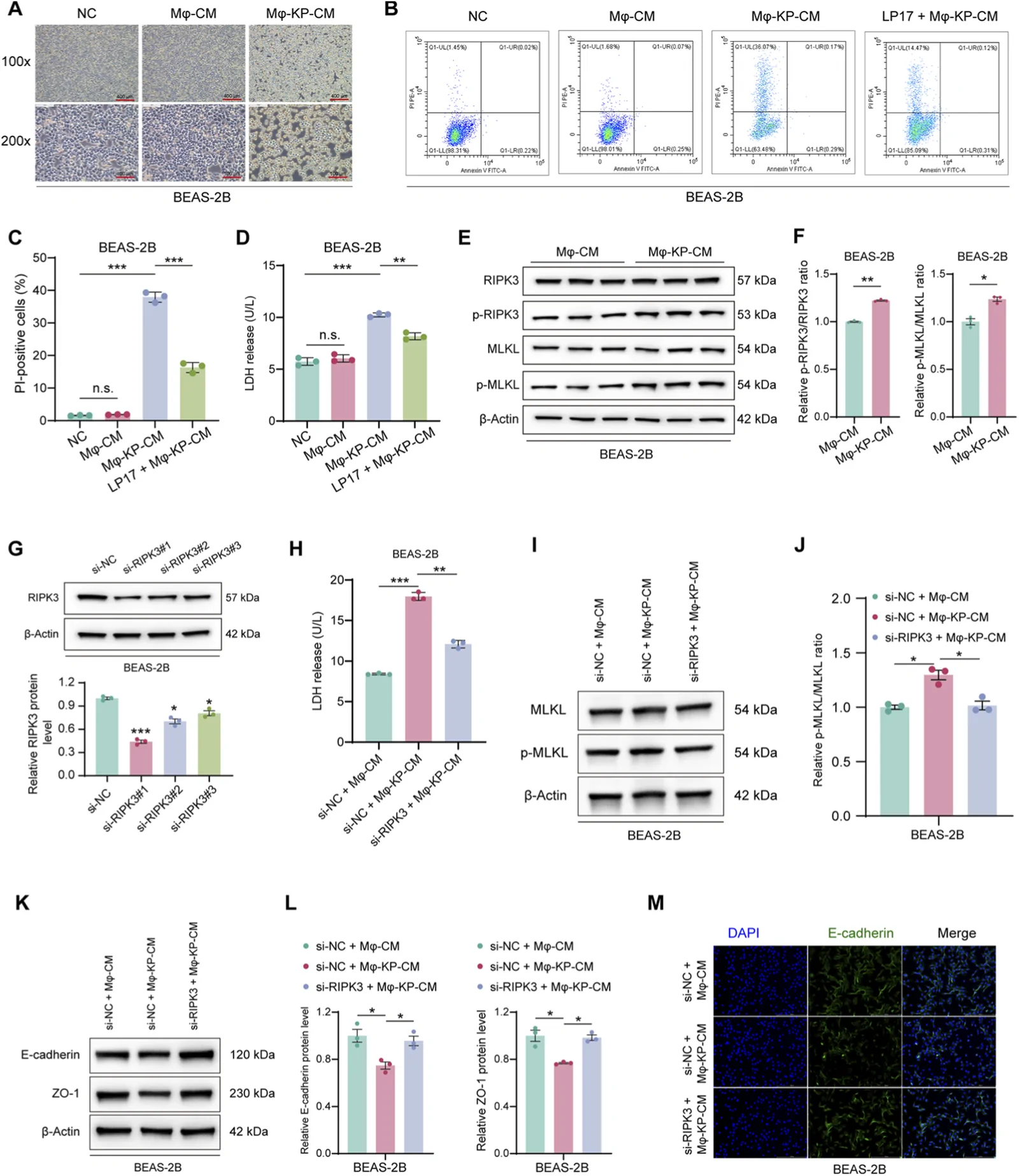
KP-stimulated macrophage-like cells induce RIPK3/MLKL-associated epithelial injury and junctional disruption in BEAS-2B cells. **(A)** Representative phase-contrast images of BEAS-2B cells exposed for 24 h to fresh medium (NC), conditioned medium from unstimulated THP-1-derived macrophage-like cells (Mφ-CM), or conditioned medium from KP-stimulated macrophage-like cells (Mφ-KP-CM). Images were acquired at ×100 and ×200 magnification; scale bars are shown in the images. **(B)** Representative Annexin V-FITC/PI flow-cytometry plots of BEAS-2B cells treated with NC, Mφ-CM, Mφ-KP-CM, or conditioned medium from LP17-pretreated, KP-stimulated macrophage-like cells (LP17 + Mφ-KP-CM). **(C)** Quantification of PI-positive BEAS-2B cells, defined as the combined upper-left and upper-right quadrants. PI positivity was interpreted as loss of plasma membrane integrity rather than as a specific marker of necroptosis. **(D)** LDH release from BEAS-2B cells after the indicated treatments. **(E)** Representative Western blots showing *RIPK3*, p-RIPK3, MLKL, and p-MLKL in BEAS-2B cells exposed to Mφ-CM or Mφ-KP-CM. **(F)** Densitometric quantification of *RIPK3* and MLKL phosphorylation, expressed as the p-RIPK3/*RIPK3* and p-MLKL/MLKL ratios. **(G)** Western blot verification and densitometric quantification of *RIPK3* knockdown in BEAS-2B cells transfected with negative-control siRNA (si-NC) or *RIPK3*-targeting siRNAs. **(H)** LDH release from si-NC- or si-RIPK3-transfected BEAS-2B cells subsequently exposed to Mφ-CM or Mφ-KP-CM for 24 h. **(I)** Representative Western blots showing MLKL and p-MLKL after *RIPK3* knockdown and conditioned-medium treatment. **(J)** Densitometric quantification of the p-MLKL/MLKL ratio. **(K)** Representative Western blots showing E-cadherin and ZO-1 after *RIPK3* knockdown and conditioned-medium treatment. **(L)** Densitometric quantification of E-cadherin and ZO-1 normalized to β-actin. **(M)** Representative immunofluorescence images showing E-cadherin localization after *RIPK3* knockdown and conditioned-medium treatment. Nuclei were counterstained with DAPI, and scale bars are shown in the images. β-Actin served as the loading control for Western blot analyses. Data are presented as mean ± SD from three independent biological experiments *P < 0.05, **P < 0.01, ***P < 0.001; n. s., not significant.

Annexin V/PI analysis showed that Mφ-KP-CM increased the proportion of PI-positive BEAS-2B cells compared with the normal-control and Mφ-CM groups. Conditioned medium from LP17-pretreated, KP-infected macrophage-like cells produced a smaller increase in PI-positive epithelial cells than Mφ-KP-CM (Figures 8B,C). Because PI positivity reflects loss of plasma membrane integrity but is not specific to a particular form of regulated cell death, these findings were interpreted as evidence of epithelial membrane injury.

Consistent with the flow-cytometric findings, Mφ-KP-CM increased LDH release from BEAS-2B cells. LP17 pretreatment of macrophage-like cells reduced the increase in LDH release induced by Mφ-KP-CM (Figure 8D). These results indicate that TREM-1 activity in KP-stimulated macrophage-like cells contributes to the production of soluble factors associated with epithelial membrane damage.

Western blotting showed that Mφ-KP-CM increased RIPK3 and MLKL phosphorylation in BEAS-2B cells compared with Mφ-CM, whereas total RIPK3 and MLKL protein levels showed no comparable increase (Figures 8E,F). Thus, macrophage-derived conditioned medium induced epithelial injury accompanied by activation of RIPK3/MLKL-associated signaling.

To examine the contribution of epithelial *RIPK3*, BEAS-2B cells were transfected with *RIPK3*-targeting siRNAs. Western blotting confirmed reduced *RIPK3* protein abundance following siRNA transfection (Figure 8G). *RIPK3* knockdown decreased Mφ-KP-CM-induced LDH release (Figure 8H), supporting the involvement of epithelial *RIPK3* in conditioned medium-associated membrane injury.

RIPK3 knockdown also reduced Mφ-KP-CM-induced MLKL phosphorylation, as reflected by a lower p-MLKL/MLKL ratio (Figures 8I,J). These findings place epithelial RIPK3 upstream of MLKL phosphorylation under the experimental conditions. Nevertheless, because MLKL was not independently inhibited or silenced, the experiments demonstrate RIPK3/MLKL-associated injury but do not fully establish MLKL-dependent necroptotic execution.

Finally, Mφ-KP-CM reduced E-cadherin and ZO-1 protein abundance in BEAS-2B cells, indicating disruption of epithelial junction-associated proteins. RIPK3 knockdown attenuated the reductions in E-cadherin and ZO-1 expression (Figures 8K,L). Immunofluorescence staining further showed weakened and discontinuous E-cadherin localization after Mφ-KP-CM exposure, whereas RIPK3 knockdown improved E-cadherin intensity and distribution (Figure 8M).

Collectively, these results demonstrate that KP-stimulated macrophage-like cells release soluble factors that induce epithelial membrane injury, RIPK3-dependent MLKL phosphorylation, and loss of junction-associated proteins. Blocking TREM-1 in macrophage-like cells or reducing RIPK3 expression in epithelial cells attenuated these effects.

## 4 Discussion

In the present study, we investigated the relationship between TREM-1-dependent myeloid inflammation and RIPK3/MLKL-associated epithelial injury in *K. pneumoniae* (KP)-induced acute lung injury. *Trem1* deficiency attenuated pulmonary histopathological injury and inflammatory mediator production and was accompanied by reduced RIPK3 and MLKL phosphorylation in lung tissues. Prophylactic administration of the RIPK3 inhibitor GSK872 produced changes in the same direction. In complementary human lung single-cell reference datasets, *TREM1* expression was predominantly localized to inflammatory myeloid populations, and a *TREM1*-high macrophage state showed predicted communication with epithelial cells through several inflammatory ligand–receptor pathways. Finally, conditioned medium from KP-stimulated macrophage-like cells induced epithelial membrane injury, RIPK3/MLKL phosphorylation, and loss of junction-associated proteins, whereas macrophage TREM-1 blockade or epithelial RIPK3 knockdown attenuated these effects. Together, these findings support an association between macrophage TREM-1 activation and RIPK3/MLKL-associated epithelial injury during bacterial lung inflammation.

TREM-1 functions as an amplifier of innate immune responses by cooperating with pathogen-recognition pathways in neutrophils and monocytes/macrophages (Bouchon et al., 2000; Bouchon et al., 2001). Previous studies have shown that TREM-1 activation enhances inflammatory cytokine production during pneumonia and experimental acute lung injury, whereas genetic or pharmacological inhibition of TREM-1 can reduce excessive pulmonary inflammation (Yuan et al., 2017; Gibot et al., 2004; Gibot et al., 2006; Dower et al., 2008; Liu et al., 2016; Weber et al., 2014; Matellan et al., 2024; Li et al., 2023; Vandestienne et al., 2021). Consistent with these observations, KP challenge increased pulmonary and soluble TREM-1 levels in our model, and *Trem1*-deficient mice exhibited reduced inflammatory mediator production and less severe histopathological injury. KP also increased TREM-1 expression, cell-surface abundance, and soluble TREM-1 release in THP-1-derived macrophage-like cells. The induction observed after both live and heat-killed KP suggests that bacterial structural components may contribute to TREM-1 activation, although the greater response to live bacteria indicates that bacterial viability or active host–pathogen interactions may provide additional stimulation.

An important issue in infection models is whether suppression of inflammatory signaling compromises antimicrobial defense. TREM-1 can contribute to pathogen recognition and immune-cell activation, but excessive TREM-1 signaling may also drive tissue-damaging inflammation. Previous evidence suggests that TREM-1 deficiency can reduce disease severity without necessarily impairing pathogen clearance in some infectious settings (Weber et al., 2014). However, bacterial burdens were not systematically measured in the present study. Therefore, the reduced pulmonary inflammation observed in *Trem1*-deficient mice cannot yet be fully separated from potential differences in bacterial replication or clearance. Future studies should quantify KP CFU in BALF, lung tissue, and blood at multiple time points to determine whether the protective phenotype reflects controlled inflammatory amplification without loss of antimicrobial competence.

RIPK3 and MLKL are central components of necroptosis-associated signaling. Following activation, RIPK3 phosphorylates MLKL, promoting MLKL oligomerization and membrane translocation, which can lead to membrane permeabilization and the release of damage-associated molecular patterns (Wang et al., 2016; Wang et al., 2014; Sun et al., 2012). RIPK3/MLKL activation has been implicated in experimental acute lung injury, pulmonary infection, and clinical ARDS (Bouchon et al., 2001; Yuan et al., 2017; Zhong et al., 2023a). In the present study, KP challenge increased pulmonary RIPK3 and MLKL phosphorylation, whereas *Trem1* deficiency reduced both phosphorylation events. Prophylactic GSK872 administration also reduced pathway activation, inflammatory mediator production, and histopathological injury. These findings suggest that RIPK3-associated signaling contributes to KP-induced lung injury and may lie downstream of, or interact with, TREM-1-dependent inflammatory amplification.

Nevertheless, RIPK3 has inflammatory functions beyond MLKL-mediated necroptosis, and phosphorylation of RIPK3 and MLKL alone does not fully establish the execution of necroptotic cell death (Yuan et al., 2017; Gibot et al., 2004; Gibot et al., 2006; Dower et al., 2008; Liu et al., 2016; Weber et al., 2014; Matellan et al., 2024; Li et al., 2023; Vandestienne et al., 2021). Similarly, PI positivity and LDH release indicate loss of membrane integrity but do not distinguish necroptosis from secondary necrosis or other lytic forms of cell death. Our data should therefore be interpreted as demonstrating RIPK3/MLKL-associated epithelial injury rather than definitively quantifying necroptosis. Direct assessment of MLKL oligomerization or membrane translocation, together with genetic or pharmacological inhibition of MLKL, would be required to establish MLKL-dependent necroptotic execution more rigorously.

The human single-cell analyses provided complementary information regarding the cellular distribution of *TREM1*. Importantly, GSE122960 and GSE135893 contain non-fibrotic and fibrotic human lung tissues rather than samples from patients with acute bacterial lung injury or ARDS. Accordingly, these datasets were used as cross-disease human lung reference cohorts. Across these datasets, *TREM1* expression was concentrated mainly in the myeloid compartment, and a *TREM1*-high inflammatory macrophage state co-expressed IL1B and TNF. This population was enriched in fibrotic lungs relative to non-fibrotic controls. These findings suggest that expansion of a *TREM1*-high inflammatory myeloid program is not restricted to acute experimental infection and may occur across pulmonary diseases characterized by inflammatory and structural remodeling. However, they do not demonstrate that the same population expands during KP-induced acute lung injury.

Pseudotime analysis placed transitional monocyte-associated cells near the selected trajectory root and *TREM1*-high inflammatory macrophages toward a distal region. This configuration is compatible with a potential transition toward an inflammatory macrophage state. Pseudotime, however, reconstructs transcriptional continuity and does not directly establish lineage origin, migration, or temporal direction. Consequently, the analysis cannot confirm that circulating monocytes differentiate into *TREM1*-high macrophages during KP infection. Lineage tracing, temporal single-cell sampling, or fate-mapping experiments would be needed to test this possibility directly.

Ligand–receptor inference further suggested that *TREM1*-high macrophages possess substantial outgoing communication potential toward epithelial cells. Predicted interactions included *TNF*–*TNFRSF1A*, *IL1B*-IL1R1, *FASLG*-FAS, *SPP1*-CD44, and *LGALS9*-CD44. Among these pathways, *TNF* signaling is particularly relevant because TNFR1 activation can engage RIPK1/RIPK3/MLKL signaling under appropriate cellular condition (Yuan et al., 2017; Zhong et al., 2023a; Matellan et al., 2024; Li et al., 2023; Rawat et al., 2020; Li et al., 2024). TREM-1 activation is also known to amplify *TNF* production during microbial and inflammatory stimulation (Shashaty et al., 2019; Pasparakis and Vandenabeele, 2015; Kaczmarek et al., 2013). Nevertheless, CellChat identifies coordinated ligand and receptor expression rather than direct ligand secretion, receptor engagement, or downstream pathway activation. The predicted *TNF*-TNFRSF1A interaction therefore generated a biologically plausible hypothesis but did not establish a causal macrophage *TNF*–epithelial TNFR1 pathway.

The conditioned-medium experiments provided functional support for soluble macrophage-derived factors as contributors to epithelial injury. Conditioned medium from KP-stimulated macrophage-like cells increased PI positivity, LDH release, RIPK3 and MLKL phosphorylation, and loss of E-cadherin and ZO-1 in BEAS-2B cells. Pretreatment of macrophage-like cells with LP17 reduced epithelial membrane injury, whereas epithelial RIPK3 knockdown reduced LDH release, MLKL phosphorylation, and junction-associated protein loss. These findings support a two-compartment model in which macrophage TREM-1 activity influences the production of soluble factors and epithelial RIPK3 contributes to the resulting injury response.

However, the present experiments did not identify which conditioned-medium component was responsible for RIPK3 activation. Although KP increased macrophage TNF-α and IL-1β release and the single-cell analysis predicted TNF–TNFRSF1A and IL1B–IL1R1 interactions, neither cytokine was individually neutralized. Recombinant TNF-α rescue after TREM-1 blockade was also not performed. Therefore, the results do not yet demonstrate that macrophage-derived TNF-α directly activates epithelial TNFR1–RIPK3/MLKL signaling. TNF-neutralizing antibodies, epithelial TNFRSF1A knockdown, and recombinant TNF-α rescue experiments would be required to establish this pathway causally.

IL-1β may contribute to a partially distinct component of the epithelial response. Previous studies have linked IL-1β signaling to increased epithelial permeability and disruption of junction-associated proteins (Rawat et al., 2020; Kaminsky et al., 2021). In the present study, KP-stimulated macrophage conditioned medium reduced E-cadherin and ZO-1 expression, and this reduction was attenuated by RIPK3 knockdown. However, IL-1β is not generally considered a canonical direct activator of the RIPK3/MLKL pathway. It is therefore premature to conclude that TNF-α and IL-1β converge directly on epithelial RIPK3/MLKL signaling. Cytokine-specific neutralization experiments may reveal that TNF-α preferentially contributes to RIPK3/MLKL-associated membrane injury, whereas IL-1β has a greater role in inflammatory barrier dysfunction (Wittekindt, 2017).

The reduction of epithelial injury after macrophage LP17 pretreatment supports the potential value of modulating TREM-1-dependent inflammation. However, LP17 was applied before bacterial exposure, and GSK872 was administered before KP challenge. The study therefore demonstrates preventive, mechanism-oriented effects rather than therapeutic efficacy after established infection or lung injury. TREM-1 also contributes to antimicrobial immune responses, creating a potential trade-off between limiting inflammatory tissue damage and preserving host defense. Accordingly, the present findings should not be interpreted as establishing a clinically applicable treatment strategy or therapeutic window.

Several limitations should be acknowledged. First, THP-1-derived macrophage-like cells and BEAS-2B cells do not fully reproduce primary alveolar macrophage and alveolar epithelial phenotypes. Second, the human datasets were derived from pulmonary fibrosis and provide cross-disease cellular context rather than infection-specific validation. Third, the conditioned-medium system does not identify the specific soluble mediator responsible for epithelial RIPK3 activation and does not reproduce direct cell contact or the complete lung microenvironment. Fourth, MLKL was not independently inhibited or silenced; therefore, the findings support RIPK3/MLKL-associated injury but do not establish MLKL-dependent necroptotic execution. Fifth, bacterial burden, physiological lung function, and survival were not assessed. Sixth, only male mice were studied, which limits assessment of sex-dependent responses. Finally, both LP17 and GSK872 were used in preventive designs, so efficacy after disease establishment remains unknown.

In conclusion, *Trem1* deficiency attenuated KP-induced acute lung injury and was associated with reduced inflammatory responses and RIPK3/MLKL activation. Human fibrosis single-cell analyses identified a *TREM1*-high inflammatory macrophage state with predicted epithelial-directed communication, while conditioned-medium experiments supported a functional contribution of macrophage TREM-1 activity and epithelial RIPK3 to membrane injury and junctional disruption. Together, the data support an interconnected inflammatory injury network, not a fully established linear TREM-1-to-RIPK3/MLKL pathway. Further validation is required before this relationship can be considered a therapeutically actionable signaling axis.

## 5 Conclusion

*Trem1* deficiency attenuated *K. pneumoniae*-induced acute lung injury and was associated with reduced inflammation and RIPK3/MLKL activation. Cross-disease human lung single-cell data and conditioned-medium experiments further support a functional relationship between macrophage TREM-1-dependent inflammatory activation and epithelial RIPK3/MLKL-associated membrane and junctional injury. These findings define a candidate intercellular injury network that requires further mechanistic and post-infection validation.

## Data Availability

The original contributions presented in the study are included in the article/Supplementary Material, further inquiries can be directed to the corresponding author.
