# Supplementary material for: TREM-1 deficiency attenuates RIPK3/MLKL-associated epithelial injury in Klebsiella pneumoniae-induced acute lung injury
Source: Front Cell Dev Biol. 2026 Oct 13;14:1974113. doi: 10.3389/fcell.2026.1974113 (PMC13710269; doi:10.3389/fcell.2026.1974113)
Supplement: Supplementary file 1 [file Table1.docx]

**Supplementary Table S1. Oligonucleotide sequences used in this study.**

| Target | Species | Sequence (5′–3′) | Application |
| --- | --- | --- | --- |
| TREM1-F | Human | CGATGTCTCCACTCCTGACTCT | qRT-PCR |
| TREM1-R | Human | CAGCAAACAGGACAGAGAAGACC | qRT-PCR |
| CD68-F | Human | CGAGCATCATTCTTTCACCAGCT | qRT-PCR |
| CD68-R | Human | ATGAGAGGCAGCAAGATGGACC | qRT-PCR |
| ITGAM/CD11B-F | Human | GGAACGCCATTGTCTGCTTTCG | qRT-PCR |
| ITGAM/CD11B-R | Human | ATGCTGAGGTCATCCTGGCAGA | qRT-PCR |
| GAPDH-F | Human | GTCTCCTCTGACTTCAACAGCG | qRT-PCR |
| GAPDH-R | Human | ACCACCCTGTTGCTGTAGCCAA | qRT-PCR |
| si-RIPK3-1 | Human | TTGGACCCAGAGCTGTTATTT | RIPK3 knockdown |
| si-RIPK3-2 | Human | GTCTCCGAGGTAAAGCATTAT | RIPK3 knockdown |
| si-NC | Human | Commercial non-targeting negative-control siRNA | Negative control |
